# Enhancing COVID-19 Vaccines Acceptance: Results from a Survey on Vaccine Hesitancy in Northern Italy

**DOI:** 10.3390/vaccines9040378

**Published:** 2021-04-13

**Authors:** Chiara Reno, Elisa Maietti, Maria Pia Fantini, Elena Savoia, Lamberto Manzoli, Marco Montalti, Davide Gori

**Affiliations:** 1 Department of Biomedical and Neuromotor Sciences, Alma Mater Studiorum—University of Bologna, 40126 Bologna, Italy; chiara.reno@studio.unibo.it (C.R.); mariapia.fantini@unibo.it (M.P.F.); marco.montalti7@studio.unibo.it (M.M.); davide.gori4@unibo.it (D.G.); 2 Emergency Preparedness Research Evaluation & Practice (EPREP) Program, Division of Policy Translation & Leadership Development, Harvard T.H. Chan School of Public Health, Boston, MA 01451, USA; esavoia@hsph.harvard.edu; 3 Department of Medical Sciences, University of Ferrara, Via Fossato di Mortara 64B, 44121 Ferrara, Italy; lmanzoli@post.harvard.edu

**Keywords:** vaccine hesitancy, COVID-19, SARS-CoV-2, vaccine hesitancy predictors, Italy, classification tree, survey, past vaccination refusal, risk perception

## Abstract

In March 2021, the coronavirus disease 2019 (COVID-19) pandemic still poses a threat to the global population, and is a public health challenge that needs to be overcome. Now more than ever, action is needed to tackle vaccine hesitancy, especially in light of the availability of effective and safe vaccines. A cross-sectional online survey was carried out on a representative random sample of 1011 citizens from the Emilia-Romagna region, in Italy, in January 2021. The questionnaire collected information on socio-demographics, comorbidities, past vaccination refusal, COVID-19-related experiences, risk perception of infection, and likelihood to accept COVID-19 vaccination. Multiple logistic regression analyses and classification tree analyses were performed to identify significant predictors of vaccine hesitancy and to distinguish groups with different levels of hesitancy. Overall, 31.1% of the sample reported hesitancy. Past vaccination refusal was the key discriminating variable followed by perceived risk of infection. Other significant predictors of hesitancy were: ages between 35 and 54 years, female gender, low educational level, low income, and absence of comorbidities. The most common concerns about the COVID-19 vaccine involved safety (54%) and efficacy (27%). Studying the main determinants of vaccine hesitancy can help with targeting vaccination strategies, in order to gain widespread acceptance—a key path to ensure a rapid way out of the current pandemic emergency.

## 1. Introduction

On 27 December 2020, less than a year after a cluster of pneumonia of unknown origin was reported in Wuhan City, in the Chinese province of Hubei, a vaccination campaign against coronavirus disease 2019 (COVID-19) started across Europe [1]. Massive efforts have been made to rapidly develop and produce vaccines to counter the spread of the disease, caused by severe acute respiratory syndrome coronavirus 2 (SARS CoV-2). It has become possible to develop a number of vaccines within a relatively short period of time due to the wide knowledge of vaccine production gained throughout the years, the use of well-established production systems, and compressed trial timelines [2]. Novel methods have been employed to increase the speed of vaccine production, such as the use of mRNA platforms [2] and by using resources from the U.S. federal government and the U.S. private sector; Operation Warp Speed (OWS) accelerated the testing, supply, development, and distribution of the vaccines in the U.S. and abroad. Furthermore, in Europe, the development and approval of COVID-19 vaccines has been accelerated thanks to the adoption of rapid review procedures, such as a rolling review, a tool to speed up the assessment of a vaccine reviewing data from ongoing studies as they become available, and the engagement of a dedicated expert task force by the European Medicines Agency (EMA) [3].

Currently, four vaccines against COVID-19 have been authorized for use in the European Union (EU) by EMA: BioNTech-Pfizer, Moderna (both based on mRNA platforms), AstraZeneca (non-replicative recombinant chimpanzee adenovirus-based vaccine), and Johnson & Johnson (non-replicating viral vector vaccine) [4]. Conditional marketing authorization has been granted after rigorous assessment of data on the quality, safety, and efficacy of these vaccines. In addition, two contracts have been executed, allowing the procurement of other vaccines once proven safe and effective, including Sanofi–GlaxoSmithKline (recombinant vaccine—protein-based antigen), and CureVac (mRNA-based vaccine), while negotiations are ongoing with Novavax (protein subunit vaccine) and Valneva (inactivated virus vaccine) [4].

However, the availability of safe and effective vaccines is not enough. In addition to the logistical and organizational issues that need to be addressed when planning and implementing a mass vaccination campaign, population acceptance towards vaccination is critical to reach adequate coverage in a population. The accelerated pace of vaccine development represents a great accomplishment for science, but can also lead to public anxiety and concerns in regards to safety issues [5], leading to vaccine hesitancy. Tackling vaccine hesitancy is essential for a successful vaccination campaign. A Strategic Advisory Group of Experts (SAGE) report from the Working Group on Vaccine Hesitancy concluded that “vaccine hesitancy refers to delay in acceptance or refusal of vaccination despite availability of vaccination services. Vaccine hesitancy is complex and context specific, varying across time, place and vaccines” [6]. Therefore, it is important to understand who the hesitant segments of the population are, and the possible reasons as to why people are hesitant, in order to develop and adopt appropriate tools and targeted communication strategies to counter such hesitancy.

Multiple investigations have been carried out to understand peoples’ willingness to take the COVID-19 vaccine. Globally, a declining trend of COVID-19 vaccination intent has been documented [7,8]. In particular, several surveys conducted in the U.S. found a number of different factors as drivers of vaccine acceptance [8]. Major population concerns are related to the safety and efficacy of the vaccines, due to their rapid approval process, and fear of side effects. Additionally, lack of trust and past experience with discrimination have been identified as determinants of vaccine hesitancy [8,9,10].

A global survey—carried out in June 2020 on a sample of 13,426 respondents across 19 countries—found that 71.5% would take a vaccine if proven safe and effective, and 48.1% would get vaccinated if their employer recommended it [5]. Men aged 18–24, and those with lower incomes, were less likely to accept the vaccine. However, besides these general results, authors observed a high heterogeneity in responses among countries, highlighting the need to understand such variation and to address community-specific concerns.

Another study carried out in the U.S. on a sample of 672 participants found a 67% overall acceptance of the COVID-19 vaccine. Males, older individuals, Asians, and college and/or graduate degree holders were more likely to accept the vaccine than their counterparts [11]. However, wide demographic and geographical variations in vaccine acceptance for COVID-19 were reported, further highlighting the need for evidence-based community communication to improve the acceptance and effectively respond to the pandemic [11].

In Italy, a survey of a random sample of 1004 adult citizens interviewed in May 2020, immediately after the national lockdown, found that 15% of respondents would probably refuse the vaccine if available, while 26% declared to be hesitant [12].

Considering the variety of situations in which vaccine hesitancy can arise, interventions should be specific to local population concerns [13].

In Italy, each region oversees the organization and delivery of health services to its residents with a level of autonomy, e.g., in providing services, in addition to the package of services established by the central government, and in organizing public health activities; the system is highly decentralized and there are variations across regions.

The aim of this study is to investigate predictors of COVID-19 vaccine hesitancy in a representative sample of the population (the Emilia-Romagna region, Italy), as a case study for other geographical contexts with similar characteristics. The study is based on a survey conducted at the beginning of the European vaccination campaign, when the Pfizer–BioNTech and Moderna vaccines were delivered to healthcare workers, and other vaccines were under review by the EMA, with the ultimate goal of informing policymakers and stakeholders on how to target and enhance their public communication strategies during the vaccination campaign.

## 2. Materials and Methods

### 2.1. Study Design and Data Collection

Our study was a cross-sectional online survey. A professional panel provider (Doxa S.p.A.) recruited a representative random sample of citizens of the Emilia-Romagna region, using a quota-based sampling strategy to ensure representativeness by socio-demographics and geographical distribution. Participant ages ranged from 18 to 70 years old among residents of the Emilia-Romagna region. The survey was implemented from January 19 to January 26, 2021. The data management of Doxa was performed in accordance with the General Data Protection Regulation of the EU.

### 2.2. Questionnaire

The questionnaire was adapted by a survey tool previously used in the U.S. [9]; the questions focused on socio-demographic characteristics, comorbidities, history of vaccination refusal, COVID-19-related experiences, perceived risk of infection, and likelihood of accepting the COVID-19 vaccination. An English version of the survey instrument can be found in the Supplementary Materials (see Supplementary Material, Questionnaire instrument).

Cognitive testing was conducted prior to full implementation. Feedback was used to revise the questionnaire. This survey was designed to be completed in approximately ten minutes.

### 2.3. Vaccine Hesitancy (Dependent Variable)

Respondents were asked about their willingness to get vaccinated if a COVID-19 vaccine was offered to them free of charge in the months ahead. Answer options were: “very likely”, “somewhat likely”, “not sure”, “not in the next two months but would consider it in the future”, “somewhat unlikely”, “very unlikely”. For the purpose of this analysis, the responses were dichotomized into two categories (1 = “Hesitant” including very unlikely/somewhat unlikely/not sure/not in the next months but I would consider it in the future; 0 = “Confident”, including very likely/somewhat likely).

### 2.4. Putative Predictors of Vaccine Hesitancy (Independent Variables)

Independent variables included: socio-demographics such as age, gender, educational level, employment status, family income, number of family members, and having a cohabiting family member older than 70. Other independent variables were a diagnosis of COVID-19, experience of income reduction due to the COVID-19 pandemic, past vaccination refusal, risk perception of contracting the disease or infecting others, and presence of comorbidities associated with a clinical risk of severe consequences from COVID-19. In particular, risk perception was measured by the level of concern of contracting COVID-19 at work or outside their work environment, and of infecting family members or friends (not at all, a little, somewhat, much concerned). Comorbidities included the health conditions most frequently associated with severe COVID-19 disease or death: diabetes, cardiovascular disease, obesity, pulmonary disease, immunocompromised status, rheumatological condition, and cancer. This variable was dichotomized to distinguish the presence or absence of comorbidities.

Moreover, in the subgroup of respondents who reported having refused a vaccination recommended by a healthcare worker, refusal reasons were investigated with a multiple choice question (see Supplementary Material). Two other multiple choice questions were used to explore the motivations needed to increase confidence in the COVID-19 vaccine among hesitant respondents.

### 2.5. Statistical Analysis

Variables were described as absolute and relative frequencies. To develop a risk perception scale, the suitability of the data for factor analysis was assessed using the Kaiser–Meyer–Olkin (KMO) measure of sampling adequacy, and evaluating uniqueness and the residual correlation matrix. Comparisons of categorical variables between hesitant and confident individuals were performed using the χ^2^ test. A multiple logistic regression analysis with backward selection was used to identify independent predictors of vaccine hesitancy. Results were reported as odds ratio (OR) and 95% confidence interval (95% CI). Multicollinearity was assessed computing the variance inflation factor (VIF). The Hosmer and Lemeshow test was used to evaluate the goodness of fit of the model. Furthermore, a classification tree analysis (CTA) based on the Chi-square Automatic Interaction Detection (CHAID) procedure was performed to identify subgroups of respondents characterized by different levels of hesitancy. The advantage of this procedure, with respect to regression models, is its ability to identify significant interactions between variables and to characterize respondents who have combinations of characteristics that make them more likely to be hesitant. An internal validation of the tree was conducted using a cross-validation approach. Analyses were carried out using Stata Statistical Software 15 (StataCorp, College Station, TX, USA) and IBM SPSS Statistics, version 21. Statistical significance was set at alpha < 0.05.

## 3. Results

### 3.1. Sample Characteristics

The sample included 1011 people (55.2% female). Mean age was 46.9 ± 11.5 ranging from 19 to 70 years old. Respondents’ characteristics are reported in Table 1. One hundred-fifty-eight respondents (15.6%) reported refusing—at least once in their lifetimes—a vaccination recommended by a healthcare worker.

**Figure 1:**
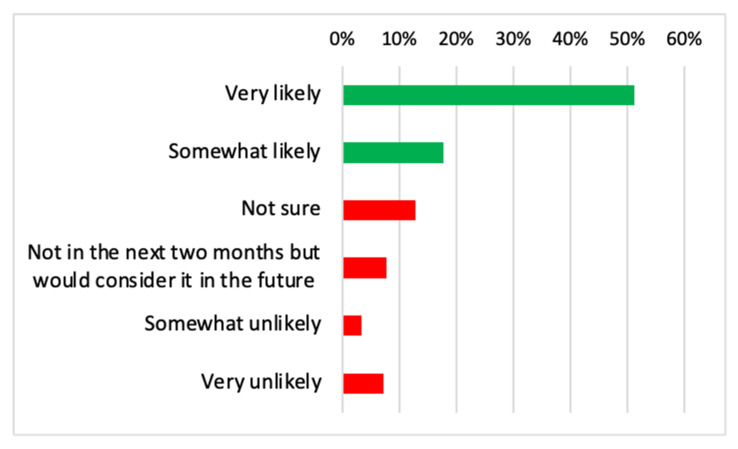
Bar chart showing participants’ response on their likelihood to get coronavirus disease 2019 (COVID-19) vaccination.

Five hundred and eighteen respondents (51.2%) reported that they would be very likely to take the COVID-19 vaccine and 17.7% (n = 179) reported to be somewhat likely to take it (Figure 1). In contrast, 7.2% respondents stated they were very unlikely and 3.4% somewhat unlikely to take the vaccine. Overall, 68.9% of the sample reported to be confident about the vaccine while 31.1% reported hesitancy.

### 3.2. Risk of Infection Perception

Seventeen percent of respondents were very concerned about getting infected at work, 19% were very concerned about contracting the disease outside the work environment, and 42% were very concerned about the possibility of infecting family members or friends (Supplementary Materials, Table S1). These three questions resulted in one factor with eigenvalue > 1, KMO = 0.71 and residual correlations < 0.1. Based on factor analysis results, a score ranging from 0 to 3.4 was obtained, with lower values indicating lower risk perception. The score was grouped in three equally-spaced classes: low risk (score < 1.2), medium risk (score 1.2–2.3), high risk (score > 2.3). Thirty-four percent of respondents were in the high-risk perception category, 47.4% in the medium risk category, and 18.5% in the low risk (Table 1).

Seventeen percent of respondents were very concerned about getting infected at work, 19% were very concerned about contracting the disease outside the work environment, and 42% were very concerned about the possibility of infecting family members or friends (Supplementary Materials, Table S1). These three questions resulted in one factor with eigenvalue > 1, KMO = 0.71 and residual correlations < 0.1. Based on factor analysis results, a score ranging from 0 to 3.4 was obtained, with lower values indicating lower risk perception. The score was grouped in three equally-spaced classes: low risk (score < 1.2), medium risk (score 1.2–2.3), high risk (score > 2.3). Thirty-four percent of respondents were in the high-risk perception category, 47.4% in the medium risk category, and 18.5% in the low risk (Table 1).

### 3.3. Predictors of Vaccine Hesitancy

We compared hesitant and confident respondents on demographic characteristics, comorbidities, previous vaccination refusal, and COVID-19-related experiences. Results indicate that the hesitant subgroup included higher proportion of respondents aged 35–44 years, and lower proportion aged <35 years and ≥55 years. The hesitant group was also characterized by a significant higher proportion of respondents with undergraduate levels of education, self-employment, and a “lower than average” family income (Table 1). Moreover, the hesitant group reported, more frequently, a previous vaccination refusal and absence of comorbidities (Table 1). As for COVID-19-related experiences, there was no difference between hesitant and confident groups with respect to COVID-19 diagnosis, while hesitant groups reported a lower level of perceived risk of infection and a higher proportion of income reduction due to restriction measures (Table 1).

Results of the logistic regression analysis are shown in Table 2. In the multivariable model, age, gender, education, family income, comorbidities, past vaccination refusal, and perceived risk of infection remained significant. No evidence of multicollinearity was found (VIFs < 5). The multiple regression model had good fit to the data (Hosmer and Lemeshow test *p* = 0.414). Female gender, undergraduate level of education, lower family income, and absence of comorbidities were significant independent predictors of hesitancy. As compared to older respondents, respondents in the age class 45–54 years and even more, those aged 35–44, had increased odds of reporting hesitancy. Conversely, there was no difference between respondents aged ≥55 years and those aged <35 years. Participants aged 35–44 years were more likely to report hesitancy even with respect to those aged 18–34 years (OR = 1.95, 95%CI: 1.20–3.15). Respondents with medium risk perception had increased odds of being hesitant compared to those with high risk perception and respondents with low risk perception were even more hesitant about taking the vaccine. Lastly, those who refused a vaccination in the past were 7.5 times more likely to report hesitancy compared to those who did not (Table 2).

### 3.4. Specific Reasons for Past Refusal

The most frequently reported reasons by the 158 individuals who refused past vaccination were “I thought the vaccine was not necessary” (37.3%) and “I was worried about side effects” (35.4%), followed by “I did not have enough information about the vaccine” (23.4%) and “I did not think the vaccine was safe” (19.6%) (Table 3). The reasons ”I did not have enough information about the vaccine” and “I did not think the vaccine was safe” were significantly associated with COVID-19 vaccine hesitancy (Table 3); those who reported lack of information and those who reported doubts on safety were more likely to be hesitant compared to those who reported other reasons.

### 3.5. Motivations Needed to Increase Confidence in COVID-19 Vaccine in Hesitant Respondents

Among the 314 hesitant respondents, the most reported motivations needed to increase their confidence in COVID-19 vaccine were “The vaccine cannot cause any immediate or long-term injury” (54.1%), “The fast production of the vaccine did not compromise its safety“ (26.1%), and “The vaccine works in protecting me from COVID-19” (26.8%) (Supplementary Materials, Table S2).

### 3.6. Classification Tree Analysis

CTA partitioned respondents into nine subgroups with different levels of hesitancy, ranging from 6.2% to 72.6% (Supplementary Materials, Figure S1). Past vaccination refusal was the key discriminating variable. Among the respondents who reported having refused a vaccination, those without comorbidities reported a higher frequency of hesitancy (72.6%) as compared to respondents with comorbidities (53.8%). Among the respondents who did not report past vaccination refusal, perceived risk of infection followed by comorbidities, family income and age generated further splits of the tree, ending up with seven subgroups. In particular, among these latter subgroups, higher levels of hesitancy were observed in the subgroup of respondents aged 35–54 years who perceived low-risk (51.8%), and in the subgroup of respondents who perceived medium-risk and reported a low family income (35.9%).

## 4. Discussion

The benefits of vaccinations are widely recognized, and vaccination is one of the most cost-effective ways to avoid infectious diseases [14]. Nonetheless, the World Health Organization (WHO) declared vaccine hesitancy as one of the top 10 global health threats. In fact, the world has witnessed a global resurgence of measles, despite the measles vaccine having a track record of over 50 years of safety and effectiveness [15]. Vaccine hesitancy is an ongoing issue and a better understanding of its determinants, within specific segments of the population, can be useful in supporting the development of effective and targeted vaccination campaigns [16].

In our survey, conducted with the aim of identifying possible predictors of COVID-19 vaccine hesitancy on a representative sample of 1011 citizens of the Emilia-Romagna region, Italy, 31.1% of respondents reported to be hesitant, a proportion similar to the one reported in other studies conducted in Europe, ranging from 20.0% (Denmark) to 38.0% (France) [17], and in the U.S., ranging from 25.0% to 43.1% [11,18,19,20].

We found that vaccine hesitancy towards COVID-19 vaccination was associated with specific characteristics of the respondents and their experiences.

First, we found that past vaccination refusal was associated with increased likelihood of hesitancy towards the COVID-19 vaccination. Confidence towards COVID-19 vaccination is higher in those who had, in the past, good attitudes toward vaccination practices, which is consistent with previous studies in Italy and the U.S., and in particular, attitude towards seasonal influenza vaccination [21,22]. In the present survey, 15.6% of all respondents refused a vaccination recommended by a healthcare worker in the past. The reasons for refusal most commonly involved the need for more information about the utility and safety of vaccines. When asked what would reassure these individuals in taking the COVID-19 vaccine, hesitant respondents said that safety and effectiveness were their major concerns, further highlighting the importance of systematically addressing these issues in communication campaigns. In a rapid literature review carried out by the London School of Hygiene and Tropical Medicine Vaccine Trust Group in 2015, “vaccine safety” was found to be the most frequently reported determinant of vaccine hesitancy, followed by “lack of information”, “low risk/severity of disease”, and “vaccines not effective” [16]. Moreover, safety concerns have been found as a key reason for hesitancy in relation to the COVID-19 vaccination [23]. It is therefore essential to address these issues in current vaccination campaigns.

We found that risk perception can influence vaccine acceptance, because individuals with medium or low risk perception were more hesitant towards the COVID-19 vaccination compared with those with higher levels of risk perception, consistent with recent studies that focus on self-perceived risk to be infected in the Italian general population during the first wave [12], or in medical staff and civilians in Israel [24]. Of note, in our study, the risk perception level was derived by considering both the level of concern of contracting COVID-19 at work or outside the work environment, and of infecting family members or friends. A high self-perceived vulnerability to the disease, but also the concern of infecting other people can lead to more acquiescent behaviors, demonstrating the importance of individual responsibility towards the community in vaccine acceptance. A study carried out in Japan also showed that willingness to protect others may have an important role in COVID-19 vaccination acceptance [25].

Respondents without comorbid conditions were more hesitant about the COVID-19 vaccination, in line with the results of a survey conducted recently in U.S. English speaking adults [22]. Subjects with pre-exiting medical conditions may possibly perceive a higher risk of severe consequences or death due to SARS-CoV-2 infection and, for this reason, might be more prone to vaccination.

Younger adults and people aged ≥ 55 years showed lower levels of hesitancy as compared to those aged 35–44 and 45–54. The U-shaped relationship between COVID-19 vaccination likelihood and age, different than for other vaccinations, has been recently shown in a study in a French population [26]. This can be explained by the fact that older people could be more likely to accept the COVID-19 vaccine because of their higher risk of severe COVID-19 disease, while younger people, less affected by the disease, possibly see the vaccine as a means to return to normal life [27] and a tool to indirectly protect older and fragile family members. This might depend on younger and older people often living together in large family settings, as it happens in Italy, as well as other Mediterranean areas.

In our study, a lower level of education and lower income were predictors of vaccine hesitancy, consistent with the findings of the previously reported study carried out in the French population after the first wave in July 2020 [26]. This relationship contrasts with the previous literature concerning child immunization, showing that more educated and wealthier parents had more concerns about vaccines safety [28,29,30,31].

In our study, the female gender was associated with higher vaccine hesitancy, in line with the current literature on vaccinations against SARS-CoV-2 [17,26,32]. The COVID-19 pandemic has, perhaps, highlighted the need to bridge the vaccine hesitancy gender gap, which historically has not been studied other than for pregnant women [33,34]. The assessment of gender roles in COVID-19 vaccine hesitancy revealed that males were more inclined to accept COVID-19 vaccines. This can be related to their higher perception of COVID-19 dangers as well as less belief in conspiracy theories [24,35]. Our findings strengthen the need to explore the role of gender in vaccine hesitancy, taking into account the epidemiological and clinical characteristics of the considered disease and different geographical and cultural contexts.

The multivariable regression model allowed identifying the role of single determinants of COVID-19 vaccine hesitancy as described so far. To take further steps in order to identify and characterize subgroups with different characteristics and levels of vaccine hesitancy, we performed a classification tree analysis. The results of this analysis may be of great importance to design targeted strategies to counteract vaccine hesitancy in specific contexts at different time points. Overall, past vaccination refusal was confirmed as the key determinant of hesitancy, further highlighting the importance of this factor. In the subgroup of individuals who refused a vaccination in the past, those who had no comorbidities had the highest level of hesitancy. Among individuals without previous vaccination refusal, the highest levels of hesitancy were found in the two subgroups with low perceived risk of infection/aged 35–54 years and with medium perceived risk of infection/lower family income, respectively. As we are facing a new infection—one that caused a pandemic—requiring the development of new vaccines, it is of fundamental importance to gain information to customize campaigns on risk communication and vaccinations offered for different settings and population groups.

It is already known that no strategy alone can address the complexity of vaccine hesitancy; therefore, coordinated efforts are necessary at a global, national, and sub-national level to address population concerns, and achieve optimal vaccine uptake [36]. Effective risk communication can be achieved with dialogue-based interventions [36], where the informational needs and concerns of populations are being identified and addressed in the communication strategy, and messages released in different contexts. In 2017, the European Centre for Disease Prevention and Control (ECDC) developed a catalogue of interventions adopted in EU/EEA (European Economic Area) countries, and in other regions, to address and measure vaccine hesitancy, providing examples of practices that could be adapted across different contexts [13]. The majority of the identified interventions were based on dialogue, communication, and information tools, with a special focus on misinformation and safety. It is important to have evidence on the overall vaccine hesitancy levels and develop activities to counteract this phenomenon; however, it is essential to have specific information, to act locally and to sustain policy makers’ decisions. Engaging with hesitant people, empowering them to ask questions, proactively listening to their concerns, providing clear, easy-to-understand, and evidence-based information [16,37] are fundamental components to encourage the acceptance of COVID-19 vaccines, but a focus should be carried out on different subgroups and their characteristics analyzed in the different contexts to better finalize information and communication campaigns. Given that concerns and acceptance towards COVID-19 vaccination can evolve with the evolution of the epidemic and with the deployment of new vaccines with different effectiveness and safety profiles for different age groups, orecurring surveys to update our knowledge and efforts to maximize vaccines uptake should be considered [38]. Indeed, a recent paper highlights that the willingness of working people in Hong Kong to accept COVID-19 vaccination declined in the third wave of the pandemic when compared to the first one [39].

The need to promote COVID-19 vaccination at a global level could offer opportunities for countries to join forces and build a solid culture of trust towards COVID-19 vaccination, as well as other vaccines, and to restore faith in this fundamental preventive measure for infectious diseases.

## 5. Limitations and Conclusions

This study has some limitations that must be considered. Cross-sectional surveys provide a picture of the period in which they are conducted. This survey was carried out immediately after the beginning of a mass vaccination campaign across Europe, but with uncertainty and dynamic changes due to delays and cuts in the delivery of doses. Moreover, this study design is not suitable to identify causal relationships. Family income was intentionally measured as perceived compared to the average instead of using quantitative measures. In accordance with the sample provider, we decided to allow respondents to self-judge their economic resources with the purpose to reach greater compliance. Similarly, comorbidities were self-reported and might be subject to reporting bias. However, we proposed predefined categories to ensure that health conditions associated with severe COVID-19 disease would be investigated. Even though our model had an acceptable goodness of fit, the set of predictors found cannot be considered exhaustive as other unknown variables may play a role in determining hesitancy.

Despite these limitations, our findings contributed to discern the multiplicity of COVID-19 vaccine hesitancy determinants, some of which are common to other vaccinations, but others are COVID-19 specific. It is important to keep in mind that different geographical and cultural contexts may play an important role: thinking globally, but acting locally can be the turning point for effective and efficient strategies. Evidence produced at different levels is needed to inform policy makers and public health professionals in their efforts to target information and communication campaigns to counteract vaccine hesitancy. Given the regional contribution in Italy to the organization of the COVID-19 vaccination campaign, this survey may represent an important piece of knowledge to enforce the need of ongoing surveys conducted at different levels and time points.

## Figures and Tables

**Table 1:** Characteristics of the sample and comparisons between confident and hesitant individuals.

Characteristics	Overall	Confident	Hesitant	*p*-Value
	(N = 1011)	(N = 697)	(N = 314)	
	N (%)	N (%)	N (%)	
**Age (years)**				0.002
18–34	154 (15.2)	117 (16.8)	37 (11.8)	
35–44	271 (26.8)	167 (24.0)	104 (33.1)	
45–54	314 (31.1)	211 (30.3)	103 (32.8)	
≥55	272 (26.9)	202 (29.0)	70 (22.3)	
**Gender**				0.235
Male	453(44.8)	321 (46.1)	132 (42.0)	
Female	558 (55.2)	376 (53.9)	182 (58.0)	
**Education**				0.013
High school or lower degree	642 (63.5)	425 (61.0)	217 (69.1)	
Bachelor’s or higher degree	369 (36.5)	272 (39.0)	97 (30.9)	
**Employment status**				0.028
Paid employed	592 (58.5)	414 (59.4)	178 (56.7)	
Self-employed	129 (12.8)	76 (10.9)	53 (17.9)	
Other	290 (28.7)	207 (29.7)	83 (26.4)	
**Family income**				0.001
Higher than average	104 (10.3)	81 (11.6)	23 (7.3)	
On average	591 (58.5)	421 (60.4)	170 (54.1)	
Lower than average	316 (31.3)	195 (28.0)	121 (38.5)	
**Number of family members**				0.413
1	112 (11.1)	72 (10.3)	40 (12.7)	
2	306 (30.3)	221 (31.7)	85 (27.1)	
3	318 (31.5)	222 (31.9)	96 (30.6)	
4	214 (21.2)	143 (20.5)	71 (22.6)	
≥5	61 (6.0)	39 (5.6)	22 (7.0)	
**Old family member (>70y)**				0.061
No	278 (27.5)	199 (28.5)	72 (22.9)	
Yes, not living together	544 (53.8)	381 (54.7)	163 (51.9)	
Yes, living together	189 (18.7)	117 (16.8)	79 (25.2)	
**Comorbidities**				<0.001
No	724 (71.6)	476 (68.3)	248 (79.0)	
One or more	287 (28.4)	221 (31.7)	66 (21.0)	
**Past vaccination refusal**				<0.001
No	853 (84.4)	644 (92.4)	209 (66.6)	
Yes	158 (15.6)	53 (7.6)	105 (33.4)	
**COVID-19 diagnosis**				0.604
No	918 (90.8)	637 (91.4)	281 (89.5)	
Yes	58 (5.7)	38 (5.4)	20 (6.4)	
Not sure	35 (3.5)	22 (3.2)	13 (4.1)	
**Income reduction due to pandemic**				0.041
No	716 (70.8)	508 (72.9)	208 (66.2)	
Yes because of quarantine	59 (5.8)	42 (6.0)	17 (5.4)	
Yes because of containment measures	236 (23.3)	147 (21.1)	89 (28.3)	
**Perceived risk of infection**				<0.001
High risk	345 (34.1)	267 (38.3)	78 (24.8)	
Medium risk	479 (47.4)	333 (47.8)	146 (46.5)	
Low risk	187 (18.5)	97 (13.9)	90 (28.7)	

*p*-values refers to *χ*^2^ test.

**Table 2:** Variables associated with vaccine hesitancy in multiple logistic regression analysis.

	Multivariable Model		
	OR	95% CI	*p*-Value
**Age (years)**	1	-	<0.001
≥55 (Reference category)	1.58	1.05–2.38	
45–54	2.31	1.51–3.54	
35–44	1.19	0.70–2.00	
18–34			
**Female**	1.39	1.02–1.89	0.038
**High school or lower degree**	1.52	1.10–2.11	0.011
**Family income**	1	-	0.044
Higher than average (Reference catetgory)	1.58	0.90–2.76	
On average	2.04	1.13–3.67	
Lower than average			
**Absence of comorbidities**	1.95	1.36–2.80	<0.001
**Past vaccination refusal**	7.52	5.02–11.3	<0.001
**Risk perception**	1	-	<0.001
High risk (Reference category)	1.47	1.04–2.08	
Medium risk	3.74	2.43–5.73	
Low risk			

*p*-value refers to the likelihood ratio test comparing the model with and without the variable.

**Table 3:** Specific reasons for past vaccine refusal: frequency of reporting and association with COVID-19 vaccine hesitancy.

Reasons for Past Vaccine Refusal	Overall (N = 158)	Confident (N = 53)	Hesitant (N = 105)	
	N (%)	N (%)	N (%)	*p*-Value
I did not think it was needed	59 (37.3)	25 (47.2)	34 (32.4)	0.070
I did not have enough information on the vaccine	37 (23.4)	4 (7.6)	33 (31.4)	0.001
I did not think the vaccine was effective	22 (13.9)	6 (11.3)	16 (15.2)	0.502
I did not think the vaccine was safe	31 (19.6)	4 (7.6)	27 (25.7)	0.007
I was worried about side effects	56 (35.4)	15 (28.3)	41 (39.1)	0.182
I had a bad experience with a previous vaccination	22 (13.9)	6 (11.3)	16 (15.2)	0.502
Logistical issues	14 (8.9)	2 (3.8)	12 (11.4)	0.110

*p*-values refer to *χ^2^* test.

## Data Availability

All data have been stored and kept by Elisa Maietti and Maria Pia Fantini, who are responsible for data analysis and appropriate keeping of data. Original data are available upon request to the corresponding author.

## Supplementary Materials

The following are available online at https://www.mdpi.com/article/10.3390/vaccines9040378/s1, File S1: Questionnaire on COVID-19 vaccination hesitancy, Table S1: Participants’ response to questions on perceived risk of infection in the overall sample and in confident and hesitant groups, Table S2: Motivations needed to increase confidence in the COVID-19 vaccine: frequency of reporting in hesitant group (N = 314), Figure S1: Classification tree showing subgroups with different levels of COVID-19 vaccine hesitancy.

## References

[1] EU Vaccination Days. http://www.politicheeuropee.gov.it/en/communication/news/european-vaccination-days-against-covid-19/.

[2] COVID-19 Vaccines: Development, Evaluation, Approval and Monitoring. https://www.ema.europa.eu/en/human-regulatory/overview/public-health-threats/coronavirus-disease-covid-19/treatments-vaccines/vaccines-covid-19/covid-19-vaccines-development-evaluation-approval-monitoring.

[3] COVID-19 Vaccines: Key Facts. https://www.ema.europa.eu/en/human-regulatory/overview/public-health-threats/coronavirus-disease-covid-19/treatments-vaccines/vaccines-covid-19/covid-19-vaccines-key-facts.

[4] Coronavirus Vaccines Strategy. https://ec.europa.eu/info/live-work-travel-eu/coronavirus-response/public-health/coronavirus-vaccines-strategy_en.

[5] Lazarus J.V., Ratzan S.C., Palayew A., Gostin L.O., Larson H.J., Rabin K., Kimball S., El-Mohandes A. (2021). A Global Survey of Potential Acceptance of a COVID-19 Vaccine. Nat. Med..

[6] MacDonald N.E. (2015). SAGE Working Group on Vaccine Hesitancy Vaccine Hesitancy: Definition, Scope and Determinants. Vaccine.

[7] COVID-19 Vaccination Intent Is Decreasing Globally. https://www.ipsos.com/en/global-attitudes-covid-19-vaccine-october-2020.

[8] Lewis J.R. (2020). What Is Driving the Decline in People’s Willingness to Take the COVID-19 Vaccine in the United States?. JAMA Health Forum.

[9] Savoia E., Piltch-Loeb R., Goldberg B., Miller-Idriss C., Hughes B., Montrond A., Kayyem J., Testa M. (2021). Predictors of COVID-19 Vaccine Hesitancy: Socio-Demographics, Co-Morbidity and Past Experience of Racial Discrimination. medRxiv.

[10] Piltch-Loeb R., Savoia E., Goldberg B., Hughes B., Verhey T., Kayyem J., Miller-Idriss C., Testa M. (2021). Examining the Effect of Information Channel on COVID-19 Vaccine Acceptance. medRxiv.

[11] Malik A.A., McFadden S.M., Elharake J., Omer S.B. (2020). Determinants of COVID-19 Vaccine Acceptance in the US. EClinicalMedicine.

[12] Graffigna G., Palamenghi L., Boccia S., Barello S. (2020). Relationship between Citizens’ Health Engagement and Intention to Take the COVID-19 Vaccine in Italy: A Mediation Analysis. Vaccines.

[13] Catalogue of Interventions Addressing Vaccine Hesitancy. https://www.ecdc.europa.eu/en/publications-data/catalogue-interventions-addressing-vaccine-hesitancy.

[14] Ten Health Issues WHO Will Tackle This Year. https://www.who.int/news-room/spotlight/ten-threats-to-global-health-in-2019.

[15] Vaccine Hesitancy Is a Global Public Health Threat. Are We Doing Enough about It?. https://www.elsevier.com/connect/vaccine-hesitancy-is-a-global-public-health-threat-are-we-doing-enough-about-it.

[16] Rapid Literature Review on Motivating Hesitant Population Groups in Europe to Vaccinate. https://www.ecdc.europa.eu/en/publications-data/rapid-literature-review-motivating-hesitant-population-groups-europe-vaccinate.

[17] Neumann-Böhme S., Varghese N.E., Sabat I., Barros P.P., Brouwer W., van Exel J., Schreyögg J., Stargardt T. (2020). Once We Have It, Will We Use It? A European Survey on Willingness to Be Vaccinated against COVID-19. Eur. J. Health Econ..

[18] Fisher K.A., Bloomstone S.J., Walder J., Crawford S., Fouayzi H., Mazor K.M. (2020). Attitudes Toward a Potential SARS-CoV-2 Vaccine: A Survey of U.S. Adults. Ann. Intern. Med..

[19] Taylor S., Landry C.A., Paluszek M.M., Groenewoud R., Rachor G.S., Asmundson G.J.G. (2020). A Proactive Approach for Managing COVID-19: The Importance of Understanding the Motivational Roots of Vaccination Hesitancy for SARS-CoV2. Front. Psychol..

[20] Reiter P.L., Pennell M.L., Katz M.L. (2020). Acceptability of a COVID-19 Vaccine among Adults in the United States: How Many People Would Get Vaccinated?. Vaccine.

[21] Caserotti M., Girardi P., Rubaltelli E., Tasso A., Lotto L., Gavaruzzi T. (2021). Associations of COVID-19 Risk Perception with Vaccine Hesitancy over Time for Italian Residents. Soc. Sci. Med..

[22] Ruiz J.B., Bell R.A. (2021). Predictors of Intention to Vaccinate against COVID-19: Results of a Nationwide Survey. Vaccine.

[23] SteelFisher G.K., Blendon R.J., Caporello H. (2021). An Uncertain Public—Encouraging Acceptance of Covid-19 Vaccines. N. Engl. J. Med..

[24] Dror A.A., Eisenbach N., Taiber S., Morozov N.G., Mizrachi M., Zigron A., Srouji S., Sela E. (2020). Vaccine Hesitancy: The next Challenge in the Fight against COVID-19. Eur. J. Epidemiol..

[25] Machida M., Nakamura I., Kojima T., Saito R., Nakaya T., Hanibuchi T., Takamiya T., Odagiri Y., Fukushima N., Kikuchi H. (2021). Acceptance of a COVID-19 Vaccine in Japan during the COVID-19 Pandemic. Vaccines.

[26] Schwarzinger M., Watson V., Arwidson P., Alla F., Luchini S. (2021). COVID-19 Vaccine Hesitancy in a Representative Working-Age Population in France: A Survey Experiment Based on Vaccine Characteristics. Lancet Public Health.

[27] Verger P., Peretti-Watel P. (2021). Understanding the Determinants of Acceptance of COVID-19 Vaccines: A Challenge in a Fast-Moving Situation. Lancet Public Health.

[28] Carpiano R.M., Polonijo A.N., Gilbert N., Cantin L., Dubé E. (2019). Socioeconomic Status Differences in Parental Immunization Attitudes and Child Immunization in Canada: Findings from the 2013 Childhood National Immunization Coverage Survey (CNICS). Prev. Med..

[29] Kempe A., Saville A.W., Albertin C., Zimet G., Breck A., Helmkamp L., Vangala S., Dickinson L.M., Rand C., Humiston S. (2020). Parental Hesitancy about Routine Childhood and Influenza Vaccinations: A National Survey. Pediatrics.

[30] Silveira M.F., Buffarini R., Bertoldi A.D., Santos I.S., Barros A.J.D., Matijasevich A., Menezes A.M.B., Gonçalves H., Horta B.L., Barros F.C. (2020). The Emergence of Vaccine Hesitancy among Upper-Class Brazilians: Results from Four Birth Cohorts, 1982–2015. Vaccine.

[31] De Cata-Preta B.O., Wehrmeister F.C., Santos T.M., Barros A.J.D., Victora C.G. (2021). Patterns in Wealth-Related Inequalities in 86 Low- and Middle-Income Countries: Global Evidence on the Emergence of Vaccine Hesitancy. Am. J. Prev. Med..

[32] Lin C., Tu P., Beitsch L.M. (2021). Confidence and Receptivity for COVID-19 Vaccines: A Rapid Systematic Review. Vaccines.

[33] Bert F., Olivero E., Rossello P., Gualano M.R., Castaldi S., Damiani G., D’Errico M.M., Di Giovanni P., Fantini M.P., Fabiani L. (2020). Knowledge and Beliefs on Vaccines among a Sample of Italian Pregnant Women: Results from the NAVIDAD Study. Eur. J. Public Health.

[34] Salmon D.A., Dudley M.Z., Glanz J.M., Omer S.B. (2015). Vaccine Hesitancy: Causes, Consequences, and a Call to Action. Am. J. Prev. Med..

[35] Sallam M., Dababseh D., Eid H., Al-Mahzoum K., Al-Haidar A., Taim D., Yaseen A., Ababneh N.A., Bakri F.G., Mahafzah A. (2021). High Rates of COVID-19 Vaccine Hesitancy and Its Association with Conspiracy Beliefs: A Study in Jordan and Kuwait among Other Arab Countries. Vaccines.

[36] Jarrett C., Wilson R., O’Leary M., Eckersberger E., Larson H.J. (2015). Strategies for Addressing Vaccine Hesitancy—A Systematic Review. Vaccine.

[37] How Can We Address Covid-19 Vaccine Hesitancy and Improve Vaccine Acceptance?. https://blogs.bmj.com/bmj/2021/02/19/how-can-we-address-covid-19-vaccine-hesitancy-and-improve-vaccine-acceptance/.

[38] Alley S.J., Stanton R., Browne M., To Q.G., Khalesi S., Williams S.L., Thwaite T.L., Fenning A.S., Vandelanotte C. (2021). As the Pandemic Progresses, How Does Willingness to Vaccinate against COVID-19 Evolve?. Int. J. Environ. Res. Public Health.

[39] Wang K., Wong E.L., Ho K.F., Cheung A.W., Yau P.S., Dong D., Wong S.Y., Yeoh E.K. (2021). Change of Willingness to Accept COVID-19 Vaccine and Reasons of Vaccine Hesitancy of Working People at Different Waves of Local Epidemic in Hong Kong, China: Repeated Cross-Sectional Surveys. Vaccines.

