# Supplementary material for: Enhancing COVID-19 Vaccines Acceptance: Results from a Survey on Vaccine Hesitancy in Northern Italy
Source: Vaccines (Basel). 2021 Apr 13;9(4):378. doi: 10.3390/vaccines9040378 (PMC8070202; doi:10.3390/vaccines9040378)

Questionnaire on COVID-19 vaccination hesitancy

Personal information

Panel information already available:

- Age (years)
- Gender
- Educational level
- Employment status
- Number of family members

1. **Have you got any close family member (father/mother/grandfather/grandmother) older than 70 years?**
    - o Yes, living together
    - o Yes, not living together
    - o No

2. **You would define your family income as…**
    - o Lower than average
    - o On average
    - o Higher than average

3. **Do you have any of the following conditions?** [select all that apply]
    - o Cancer
    - o Immunocompromised state due to therapy or disease
    - o Obesity
    - o Diabetes (type 1 or 2)
    - o Cardiovascular disease
    - o Pulmonary disease
    - o Rheumatological condition

Personal experiences

4. **If you have refused a vaccine in the past that was recommended to you by a healthcare worker - what was/were the reason(s)?** [check all reasons that applied to that situation]
    - o I never refused a vaccine recommended by a healthcare worker
    - o Did not think it was needed
    - o Did not have enough information on the vaccine
    - o Did not think the vaccine was effective
    - o Did not think the vaccine was safe
    - o I was concerned about side effects
    - o I had a bad experience with a previous vaccination
    - o Did not know where to get vaccination
    - o Other logistic problems

5. **Have you had COVID-19?**
    - o I had suspected symptoms but I didn't verify with a doctor and/or specific exams
    - o No
    - o Yes with no symptoms
    - o Yes with mild symptoms
    - o Yes with severe symptoms

6. **Have you lost your job or had an income reduction due to COVID-19 pandemic?**
   - o No
   - o Yes, lost my job or had a salary reduction due to quarantine period
   - o Yes, lost my job or had a salary reduction due to restriction measures

7. **How concerned are you of ...**

	Not at all concerned	A little concerned	Somewhat concerned	Very concerned
Contracting COVID-19 at work?<br>(For example: office and other work settings that are not your home)				
Contracting COVID-19 outside of work?<br>(For example: at the grocery store, when you are using transportation, or in other aspects of your daily life)				
Infecting your family or friends with COVID-19?				

Vaccination likelihood

8. **A COVID-19 vaccine has already been approved. If you were offered to get the vaccine in the next months - at no cost for you- how likely are you to take it?**
   - o Very likely
   - o Somewhat likely
   - o I am not sure
   - o Somewhat unlikely
   - o Very unlikely
   - o I would not take it within the next two months but I might reconsider it in the future

9. **What would be important for you to know to make you more confident in the COVID-19 vaccine?** [Select up to 3 options]
   - o The fast production of the vaccine did not compromise its safety
   - o Agencies approving the vaccines are following strict rules
   - o My risk of getting sick with COVID-19 is bigger than the risk of side effects from the vaccine
   - o The vaccine cannot cause any immediate or long term injury
   - o It is impossible to get COVID-19 or any other disease from the vaccine itself or its components
   - o The vaccine works in protecting me from COVID-19
   - o The vaccine works in stopping the transmission of COVID-19 from one person to another
   - o Health agencies and WHO recommend the vaccine and agree it is safe
   - o I do not need any other information
   - o Other - please specify______________________

10. **What else would be important for you to know to make you more likely to take the COVID-19 vaccine?** [Select up to 3 options]
   - o Once vaccinated I will be able to live my life with no restrictions
   - o Those with concerns about the vaccine have opportunities to share their opinions with the public
   - o Pharmaceutical companies will not make large profits from the vaccine
   - o Everybody will have equal access to the vaccine regardless of income or race
   - o I will be free to choose if I get the vaccine or not with no consequences
   - o There are no other reasons why so many people are sick (i.e. 5G technology or other unknown reasons)

Supplementary Results

Table S1. Participants' response to questions on perceived risk of infection in the overall sample and in confident and hesitant groups.

	**Overall (N=1011)** N (%)	**Confident (N=697)** N (%)	**Hesitant (N=314)** N (%)
**How concerned are you about contracting COVID-19 at work?**			
Not concerned	182 (18.0)	101 (14.5)	81 (25.8)
A little concerned	308 (30.5)	215 (30.9)	93 (29.6)
Somewhat concerned	346 (34.2)	256 (36.7)	90 (28.7)
Very concerned	175 (17.3)	125 (17.9)	50 (15.9)
**How concerned are you about contracting COVID-19 outside of work?**			
Not concerned	88 (8.7)	39 (5.6)	49 (15.6)
A little concerned	298 (29.5)	195 (28.0)	103 (32.8)
Somewhat concerned	434 (42.9)	315 (45.2)	119 (37.9)
Very concerned	191 (18.9)	148 (21.2)	43 (13.7)
**How concerned are you about infecting your family or friends with COVID-19?**			
Not concerned	56 (5.5)	25 (3.6)	31 (9.9)
A little concerned	151 (14.9)	86 (12.3)	65 (20.7)
Somewhat concerned	376 (37.2)	259 (37.2)	117 (37.3)
Very concerned	428 (42.3)	327 (46.9)	101 (32.2)

Table S2. Motivations needed to increase confidence in the COVID-19 vaccine: frequency of reporting in hesitant group (N=314).

	**N (%)**
**Specific motivations about vaccine**	
The vaccine cannot cause any immediate or long-term injury	**170 (54.1)**
The vaccine works in protecting me from COVID-19	**84 (26.8)**
The fast production of the vaccine did not compromise its safety	**82 (26.1)**
The vaccine works in stopping the transmission of COVID-19 from one person to another	51 (16.2)
My risk of getting sick with COVID-19 is bigger than the risk of side effects from the vaccines	51 (16.2)
I do not need other information	46 (14.7)
Agencies approving the vaccines are following strict rules	41 (13.1)
It's impossible to get the COVID-19 or any other disease from the vaccine itself or its components	37 (11.8)
Health agencies such as WHO recommended the vaccine and say it is safe	22 (7.0)
Other	8 (2.6)
**Other motivations**	
I will be free of choose if I get the vaccine or not with no consequences	**172 (54.8)**
Once vaccinated I will be able to live my life with no restrictions	**146 (46.5)**
Everybody will have equal access to the vaccination regardless of income and race	80 (25.5)
Pharmaceutical companies will not make large profits from the vaccine	71 (22.6)
Those with concerns about the vaccine have the opportunity to share their opinion with the public	44 (14.0)
There are no other reasons why so many people are sick (i.e. 5G technology, other unknown reasons)	13 (4.1)

Figure S1. Classification tree showing subgroups with different levels of COVID-19 vaccine hesitancy.

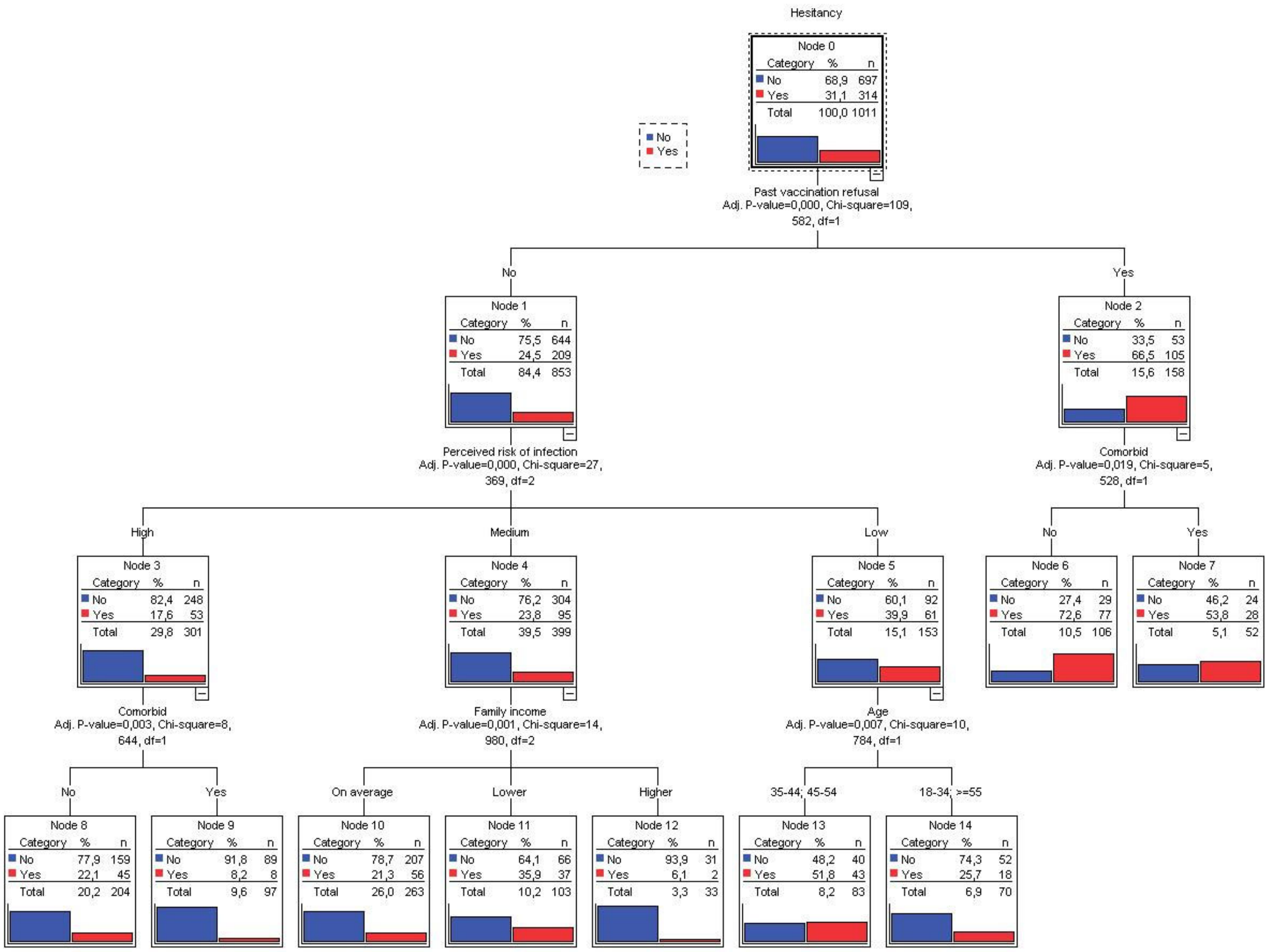

Supplement: Supplementary file 1 [file vaccines-09-00378-s001.pdf]
